# Editorial: Oral inflammation as an emerging risk factor for cardiovascular health

**DOI:** 10.3389/froh.2025.1748994

**Published:** 2025-12-03

**Authors:** M. Cardisciani, S. Di Nicolantonio, D. Pietropaoli

**Affiliations:** 1 Department of Life, Health & Environmental Sciences, University of L’Aquila, L’Aquila, Italy; 2 Center of Oral Diseases, Prevention and Translational Research - Dental Clinic, L’Aquila, Italy; 3 Department of Medicine, Case Western Reserve University, School of Medicine, Cleveland, OH, United States; 4 Department of Physical and Chemical Science, University of L’Aquila, L’Aquila, Italy

**Keywords:** oral inflammation, cardio-oral axis, cardiovascular disease, cardiovascular prevention, oral disease and systemic disease

Independent of traditional risk factors, oral diseases have recently been recognized as a non-negligible contributor to the development of cardiovascular diseases (CVDs), which are the leading cause of death globally. Poor oral health, coupled with immune dysregulation and unhealthy lifestyle habits, triggers gum inflammation that can extend beyond the oral cavity and fuel systemic inflammation. Oral pathogens originating from inflamed periodontal tissues may enter the bloodstream, disseminate and contribute to endothelial dysfunction and atheroma formation. Locally produced inflammatory mediators such as IL-6, TNF-α, and IL-1β further stimulate hepatic synthesis of acute-phase proteins, including CRP, thereby promoting atherosclerosis progression. Among oral diseases, periodontitis and dental caries remain the most prevalent worldwide, both capable of inducing oral dysbiosis and chronic immune activation. Notably, the perspective on chronic periodontitis has shifted: it is now recognized as a systemic inflammatory disease, moving beyond the former classification as merely localized oral condition.

Conversely, non-surgical periodontal therapy (NSPT) and intensive professional oral hygiene have been shown to lower systemic inflammation and reduce blood pressure in pre-hypertensive, hypertensive, and treatment-resistant individuals (1, 2). These benefits may persist over time and can potentially contribute to the attenuation of atherosclerosis progression. Such evidence strengthens the concept that maintaining oral health represents a cost-effective, non-pharmacological strategy for managing hypertension and reducing cardiovascular risk.

The aim of this Research Topic was to deepen understanding of the molecular and clinical links between oral inflammation and cardiovascular diseases. Building on this premise, the contributions included in this issue provide complementary insights that collectively reinforce the cardio-oral connection. Roberts et al., for instance, explored the association between dental caries and cardiovascular events using data from both the NHANES and DRDR databases. Their analysis revealed that higher DMFT/DMFS scores correlated with a history of cardiovascular events, even after adjustment for traditional risk factors. Interestingly, the association was stronger in the NHANES cohort, which included non-dental care seekers. Although the corss-sectional design and the absence of confounders such as BMI, diet, and physical activity limit causal inference, the findings underscore the importance of integrating medical and dental datasets in large-scale population studies to fully capture the oral-systemic interface.

Continuing this line of evidence, Church et al. emphasized the critical role of oral health awareness in cardiovascular disease prevention. Their review showed that hospitalized cardiac patients often exhibit poor adherence to oral hygiene practices, with fewer than half reported regular flossing. Educational programs (digitally or face-to-face) significantly improved both oral hygiene behaviors and postoperative cardiovascular outcomes. These results suggest that incorporating oral health education into patient management could serve as an effective secondary prevention strategy; however, a key knowledge gap remains: none assessed changes in systemic inflammatory markers.

Addressing this gap, Usmani et al. conducted a scoping review of 12 studies that linked improvements in oral health improvements to reductions in inflammatory biomarkers and CVD mortality. Specifically, non-surgical periodontal therapy (NSPT) was associated with decreases in CRP, cholesterol, and triglycerids in cardiovascular patients. Additionally, combining NSPT with good oral hygiene resulted in a halving of Staphilococcus Aureus infections and coronary heart disease mortality. Despite these clear benefits, the authors stressed the need to implement and standardize programs designed to empower vulnerable, high-risk patients to improve their oral health for better overall systemic health. In conclusion, this review reinforces the concept that oral health should be integrated into broader CVD prevention frameworks, encouraging interdisciplinary collaboration between dental and medical practitioners to reduce the global burden of non-communicable diseases.

Finally, Molina et al., provided robust clinical evidence trough a triple-blinded randomized clinical trial assessing the effects of NSPT on cardiovascular and oral outcomes in patients with chronic periodontitis and established cardiovascular disease. Over a six-month period, both supragingival scaling (Step 1) and complete subgingival instrumentation (Step 2) improved periodontal parameters, with Step 2 yielding greater reductions in bleeding on probing and probing depth. Interestingly, both protocols significantly reduced carotid intima-media thickness (cIMT), though neither influenced endothelial function as measured by flow-mediated dilation (FMD). These results, consistent with recommendations from the European Federation of Periodontology and the World Heart Federation, confirm that periodontal therapy is safe and beneficial for vascular health, even when initiated as early as three months after an acute cardiovascular event.

Collectively, the studies featured in this Research Topic provide compelling evidence of the cardio-oral connection. Maintaining periodontal health is no longer a purely dental matter but a fundamental component of systemic disease prevention. Despite this increasing body of evidence, oral health promotion remains insufficiently addressed within cardiovascular care protocols. Integrating dental assessment and preventive oral therapy into routine cardiology practice could be a simple yet highly effective strategy to mitigate systemic inflammation and improve vascular outcomes.

## References

[1] Cardisciani M Di Nicolantonio S Altamura S Ortu E Del Pinto R Pietropaoli D. Temporal dynamics of early inflammatory markers after professional dental cleaning: a meta-analysis and spline-based meta-regression of TNF-α, IL-1β, IL-6, and (hs)CRP. Front Immunol. (2025) 16:1634622. 10.3389/fimmu.2025.1634622 40948802 PMC12423065

[2] Czesnikiewicz-Guzik M Osmenda G Siedlinski M Nosalski R Pelka P Nowakowski D Causal association between periodontitis and hypertension: evidence from Mendelian randomization and a randomized controlled trial of non-surgical periodontal therapy. Eur Heart J. (2019) 40(42):3459–70. 10.1093/eurheartj/ehz646 31504461 PMC6837161

